# Evaluation of the efficacy of *Abelmoschus manihot* (L.) on diabetic nephropathy by analyzing biomarkers in the glomeruli and proximal and distal convoluted tubules of the kidneys

**DOI:** 10.3389/fphar.2023.1215996

**Published:** 2023-08-01

**Authors:** Hongmei Yu, Mei Wang, Jingshi Yu, Haitao Tang, Qing Xu, Ning Cheng, Xiaoxiao Luo, Yurong Wang, Haitao Ge, Lei Qiang, Wei Tang, Harvest F. Gu

**Affiliations:** ^1^ Laboratory of Molecular Medicine, School of Basic Medicine and Clinical Pharmacy, China Pharmaceutical University, Nanjing, China; ^2^ Suzhong Pharmaceutical Research Institute, Nanjing, China; ^3^ State Key Laboratory of Natural Medicines, School of Basic Medicine and Clinical Pharmacy, China Pharmaceutical University, Nanjing, China; ^4^ Department of Endocrinology, Islet Cell Senescence and Function Research Laboratory, Nanjing Medical University Affiliated Geriatric Hospital/Jiangsu Province Geriatric Hospital, Nanjing, China

**Keywords:** *Abelmoschus manihot* (L.), biomarker, diabetic nephropathy, Huangkui capsule, type 2 diabetes

## Abstract

**Introduction:** As a traditional Chinese medicine*, Abelmoschus manihot* (L.) in the form of Huangkui (HK) capsule has been used as a medication for kidney diseases, including diabetic nephropathy (DN), in China. The most significant effect of HK capsule treatment in kidney diseases is the reduction of albuminuria and proteinuria. To evaluate the efficacy of HK capsule in the regression of DN, in the current study, we analyzed the biomarkers in the glomerulus and proximal and distal convoluted tubules in the kidneys of db/db mice, the animal model for type 2 diabetes and DN.

**Methods:** Huangkui capsules (0.84 g/kg/d) or vehicle were administered daily via oral gavage for 4 weeks in db/db mice. Urinary albumin-to-creatinine ratio and blood glucose levels were measured during the whole experimental period. Five biomarkers in the glomerulus and proximal and distal convoluted tubules in the kidneys were selected, namely, col4a3, slc5a2, slc34a1, slc12a3, and slc4a1, and their activities at mRNA and protein levels before and after HK capsule treatment were analyzed by real-time RT–PCR and immunohistochemistry.

**Result and discussion:** After HK capsule treatment for 4 weeks, the urinary albumin-to-creatinine ratio in db/db mice was found to be significantly decreased. The activities of col4a3, slc5a2, slc34a1, slc12a3, and slc4a1 in the kidneys were upregulated in db/db mice prior to the treatment but downregulated after HK capsule treatment. Further analyses of the fields of whole kidney tissue sections demonstrated that the number of nephrons in the kidneys of db/db mice with HK capsule treatment was higher than that in the kidneys of db/db mice without HK capsule treatment. Thereby, the current study provides experimental evidence confirming the medical efficacy of *A. manihot* in the reduction of albuminuria and proteinuria, suggesting that *A. manihot* may have pharmacological efficacy in the regression of the development of type 2 diabetes-DN.

## 1 Introduction

The latest figures released by the International Diabetes Federation (IDF) show that 537 million (1 in 10) adults now live with diabetes worldwide (IDF, 2021). In China, the prevalence of diabetes reported in 2017 was 11.2% (Li P. et al., 2020), while the true incidence is likely to be higher than this number. *Type 2 diabetes* (T2D) is the *most* common sub*type* of *diabetes,* accounting for around 90% of all *diabetes* cases. Diabetic nephropathy (DN) is the most common cause of chronic kidney disease and a leading cause of end-stage renal disease (ESRD). Based on the pooled data from 54 countries, more than 80% of ESRD cases are caused by DN, hypertension, or a combination of both, and ESRD patients need to be treated with kidney dialysis or transplantation to improve their quality of life (Thomas et al., 2015; Doshi et al., 2017; Koye et al., 2018; Selby et al., 2020). Therefore, the health burden brought by DN is a major challenge to individuals, families, and society. Effective therapies for the reduction of proteinuria and regression of development are of critical importance in DN.

The molecular mechanism of DN involves an interplay of hemodynamic and metabolic pathways, providing many potential targets for novel drug therapies. The vasoactive pathways mainly include activation of the renin–angiotensin–aldosterone system (RAS), endothelin-1, and sodium–glucose cotransporter 2 (SGLT2, also known as solute carrier family 5 member 2, SLC5A2). The metabolic pathways in DN are manifested as an increase in mitochondria and reactive oxygen species, NADPH oxidase, transcription factors, advanced glycation and the end products, protein kinase C, aldose reductase, JAK-STAT, and autophagy (Warren et al., 2019). For the last 2 decades, the management methodology of DN has been risk factor control and renin–angiotensin system inhibition in the vasoactive pathways (Brenner et al., 2001; Lewis et al., 2001; Parving et al., 2001). Recent evidence has supported a reno-protective effect of SGLT2 inhibition and glucagon-like peptide 1 receptor agonists in addition to their hypoglycemic properties (Park et al., 2007; Fioretto et al., 2016; Cowie et al., 2020; DeFronzo et al., 2021).

In recent years, researchers have undertaken genetic association studies of DN for better understanding its molecular mechanisms (Gu, 2019). A genome-wide association study (GWAS) has demonstrated that the *COL4A3* gene has a susceptivity risk to DN (Salem et al., 2019). This gene encodes the collagen type IV α3 chain, which is one of the major components of the glomerular basement membrane (GBM) and a multimeric protein composed of three alpha subunits (α3, α4, and α5). Based on its significance in the GBM morphologically and functionally, this gene can serve as a biomarker for the glomeruli in the kidneys (Naylor et al., 2021). Furthermore, solute carriers (SLCs) are a family of membrane proteins and found to be involved in the transport of nutrients, metabolites, xenobiotics, and drugs (Ferrada et al., 2022). Most of them are expressed in the kidneys and responsible for much of the transport of ions and organic molecules along the renal tubules (Park et al., 2018; Wu et al., 2022). Solute carrier family 12 member 3 (*SLC12A3*) mediates Na^+^ and Cl^−^ reabsorption in the distal convoluted tubule (DCT) of the kidneys, and this gene encodes a sodium-chloride cotransporter (Li et al., 2022). Previously, we demonstrated the structural abnormality of the kidney pronephric distal duct at the 1-cell stage in zebrafish after the morpholino knockdown of slc12a3 in zebrafish ortholog (Abu Seman et al., 2014). Furthermore, we found that the single-nucleotide variant Arg913Gln in the *SLC12A3* gene is associated with DN in T2D patients (Abu Seman et al., 2014). SLC5A2 is highly expressed in the proximal convoluted tubule (PCT) of the kidneys. As a target, SGLT2 inhibitors act on the proximal tubules of the kidneys and have efficacy in the management of T2D and cardiovascular and renal safety (Park et al., 2007; Fioretto et al., 2016; Cowie et al., 2020; DeFronzo et al., 2021). Recently, we performed single-cell RNA sequencing analyses in the kidneys of db/db mice and predicted a list of biomarkers in the PCT and DCT of the kidneys (Wu et al., 2022).

Traditional Chinese medicine (TCM) is perceived to be a cost-efficient alternative. In recent years, accumulating evidence has demonstrated that treatment of DN using TCM is not only clinically valuable but also supported by experimental data from basic medicine (Wen et al., 2017; Lu et al., 2019; Zhang et al., 2019). Huangkui (HK) capsule, as a TCM, is made from the ethanol extract of flowers in *Abelmoschus manihot* (L.) medic. and currently used as a medication for kidney diseases, including DN (Li et al., 2021). In the clinic, progressive albuminuria and proteinuria are important predictors of DN. Indeed, albuminuria and proteinuria are closely associated with the incidence of the mortality, renal damage, and cardiovascular, cerebrovascular, and peripheral arterial diseases in patients with T2D and DN (Chan et al., 1995; Gerstein et al., 2001; Hemmelgarn et al., 2010). From 2014 to 2022, four multicenter randomized controlled clinical trials have been conducted, and the efficacy and safety of *A manihot* (L.) for primary glomerular disease, IgA nephropathy, and DN have been reported (Zhang et al., 2014; Li et al., 2017; Li et al., 2020b; Zhao et al., 2022). The most significant effect of HK capsule therapy among patients with kidney diseases, including DN, is of the reduction of albuminuria and proteinuria. However, it is still not fully understood whether HK capsule has effects on the regression of development in kidney diseases such as DN.

A mammalian kidney consists of approximately one million nephrons at maturity. The number of nephrons in the kidney can only be gradually decreased and never increase over a lifetime (Wallace, 1998). Therefore, it is a challenge that once DN is established, its progression can only be reversed by therapeutic approaches. In China, the incidence and prevalence of diabetes have dramatically increased over the past decade. The estimated number of patients with DN in China has reached 24.3 million (Zhang et al., 2016; Yang et al., 2020). Furthermore, HK capsule is clinically used to treat DN in China. Although the efficacy of HK capsule in the reduction of albuminuria and proteinuria is significant, our knowledge to understand its pharmacological efficacy at molecular levels is still limited. In the current study, we performed experiments in db/db mice, which are animal models for T2D and DN (Sharma et al., 2003). We first repeated the experiment to confirm the effects of HK capsule in reducing albuminuria and proteinuria. We then comparatively analyzed the functional activity of five biomarkers, col4a3, slc5a2, slc34a1, slc12a3, and slc4a1, located in the glomeruli and PCT and DCT of kidneys between the db/db mice with and without HK capsule treatment. Finally, we assessed the impact of HK capsule treatment on the regression of DN development. Data from the current study may provide experimental evidence to improve our understanding of the therapeutic value of *A. manihot* in DN.

## 2 Materials and methods

### 2.1 Animals and treatment

Db/db (BKS.Cg-Dock7m +/+ Leprdb/J) mice are commonly used as animal models for T2D and DN (Sharma et al., 2003). In the current study, db/db and C57BL/6J mice (8-week-old males) were purchased from the animal experimental center of Nanjing University (Nanjing, China). All mice were maintained in a specific pathogen-free (SPF) barrier environment and housed with an alternating 12-h light/12-h dark cycle under controlled temperature (22°C ± 2°C) and humidity (50% ± 10%), with *ad libitum* access to standard chow and water at the animal experiment center, Xuanwu Campus, China Pharmaceutical University (CPU).

After 7 days of adaptation, db/db mice were divided into the DN modeling (*n* = 12) and HK capsule treatment (*n* = 12) groups. C57BL/6J mice were non-diabetic and considered as the negative control group (Cont, *n* = 6). Blood glucose levels were measured weekly. Urine samples were collected with a metabolic cage (DXL-XS, Fengshi, Suzhou, China) for 24 h once a week. Microalbuminuria (MAU) and creatinine (Cr) levels were determined by using ELISA quantitative kits (Elabscience Biotechnology, China) with sandwich ELISA and competitive ELISA principles, respectively. The db/db mice with DN were diagnosed when blood glucose levels were ≥16.7 mmol/L and the UACR value was ≥200 mg/g for two consecutive days. All experiments were performed in accordance with the guidelines of the Declaration of Helsinki and approved by the ethics committee of China Pharmaceutical University (approval code: 2019-08-0003 and approval date: 2019-08-26).

HK capsule, as a traditional Chinese medicine, has been approved by the State Food and Drug Administration (Z19990040), China. This drug is made with powder extracted from *A. manihot* (L.) and produced by Suzhong Pharmaceutical Group Co., Ltd. (Taizhou, China) (Li et al., 2021). One capsule contains 0.43 g of *A. manihot* extract. The quality of HK capsules has been examined by fingerprint analysis with high-performance liquid chromatography, as previously reported (Lai, Zhao, & Liang, 2006; Lai, Liang, Zhao, & Wang, 2009; Guo et al., 2015). In the current study, HK capsule was dissolved in distilled water and freshly prepared as an HK suspension for use. Because HK capsule is clinically used to treat a patient with DN (body weight 60 kg) at a dose of 7.5 g/day, the dose of 0.84 g/kg/day was then administered to the db/db mice in the HK group. The administration period was 4 weeks. Blood and kidney tissue samples were collected for the analyses of various indicators.

### 2.2 Hematoxylin and eosin staining analysis

Db/db mice were anesthetized with 1% sodium pentobarbital. The kidneys were removed by cardiac perfusion with PBS and placed in formaldehyde tissue fixative. The fixed kidney tissues were embedded in paraffin, and the blocks were sectioned at 4 μm with the HistoCore BIOCUT (Leica Biosystems, Germany). The sections were then stained with H&E (Baso Diagnostics, Zhuhai, China) according to the standard procedures and finally mounted on a CX23 light microscope (Olympus, Japan) for analysis. As we have recently reported (Wu et al., 2022), by using the Image-Pro Plus (version 6.0.0260) software, in the current study, 4–8 fields of H&E-stained sections from each animal were randomly selected for semi-quantification of the glomerular area, interstitial area of the glomerulus, and ratio of the vacuolar and staining areas.

### 2.3 Selection of markers in the glomerulus and proximal and distal convoluted tubules

DN is a complex disease. In its pathogenesis, the genetic and environmental factors are interplayed. Based on the information from public genome databases and pathophysiological and genetic association studies, we selected five biomarkers in the glomerulus and proximal and distal convoluted tubules to evaluate the effects of HKC in DN. The locations and biological functions of these markers in kidneys are represented in Supplementary Table S1.

### 2.4 Immunohistochemical staining and HALO analyses

Kidney sections were boiled in citrate buffer (10 mM, pH 6.0) for 20 min for antigen retrieval and then blocked in 10% normal goat serum and incubated with a primary antibody overnight at 4°C. In the current study, the used primary antibodies include col4a3 (1:50 diluted, orb313870, Biorbyt Biologicals, China), slc5a2 (1:100 diluted, NBP1-92384, Novus Biologicals, China), slc34a1 (1:100 diluted, orb499592, Biorbyt Biologicals, China), slc12a3 (orb100812, Biorbyt Biologicals, China), and slc4a1 (1:100 diluted, orb324433, Biorbyt Biologicals, China). After that, the sections were incubated with biotinylated secondary antibody (PV6001, Borui Biotechnology Co., Ltd.) for 1 h at room temperature and then developed with 3,3′-diaminobenzidine (DAB, 1:20) chromogenic assay for 30 s. The field of whole kidney sections was scanned with a fluorescence scanner (VS200, Olympus, Tokyo, Japan), and the DAB-positive cells in each image were analyzed via HALO 2.0 area quantification algorithm (Indica Labs, Corrales, NM, United States). The glomerular number was analyzed and accounted based on the structural morphology by the computer program and repeated manually.

### 2.5 Real-time RT–PCR

According to the manufacturer’s protocol, total RNAs were extracted from kidney tissues and converted into cDNA by using a FastPure cell/tissue kit and a HiScriPt-Ill 1st strand cDNA synthesis kit(Vazyme Biotech Co., Ltd. Nanjing, China), respectively. To detect the gene expression of *col4a3, slc5a2, slc34a1, slc12a3,* and *slc4a1* at mRNA levels, the quantitative PCR experiments were performed with ChamQ SYBR Color qPCR Master Mix (Vazyme) in the CFX Connect Real-Time PCR system (Bio-Rad System, United States). The primers used are summarized in Supplementary Table S2, and *GAPHD* was used as an internal reference. Real-time RT–PCR was carried out with initial DNA double-strand denaturation at 94°C for 5 min, and the cycling conditions (10 s at 95°C, 10 s at 60°C, and 30 s at 72°C) were 45 cycles. The analysis was relatively quantified to *GAPHD,* and the delta cycle-threshold (Ct) values in each sample were averaged by using the Ct method. The experiments for the detection of each gene were performed in duplicate to ensure amplification integrity.

### 2.6 Statistical analysis

To test the statistical differences among the groups, one-way analysis of variance (ANOVA) and Bonferroni *post hoc* analysis were carried out by using GraphPad Prism 5.0 software (GraphPad Software Inc., La Jolla, United States). The values were expressed as the mean SEM. The *p*-value < 0.05 was considered statistically significant.

## 3 Results

### 3.1 Confirmation of the efficacy of HK capsule on the reduction of albuminuria and proteinuria

**FIGURE 1:**
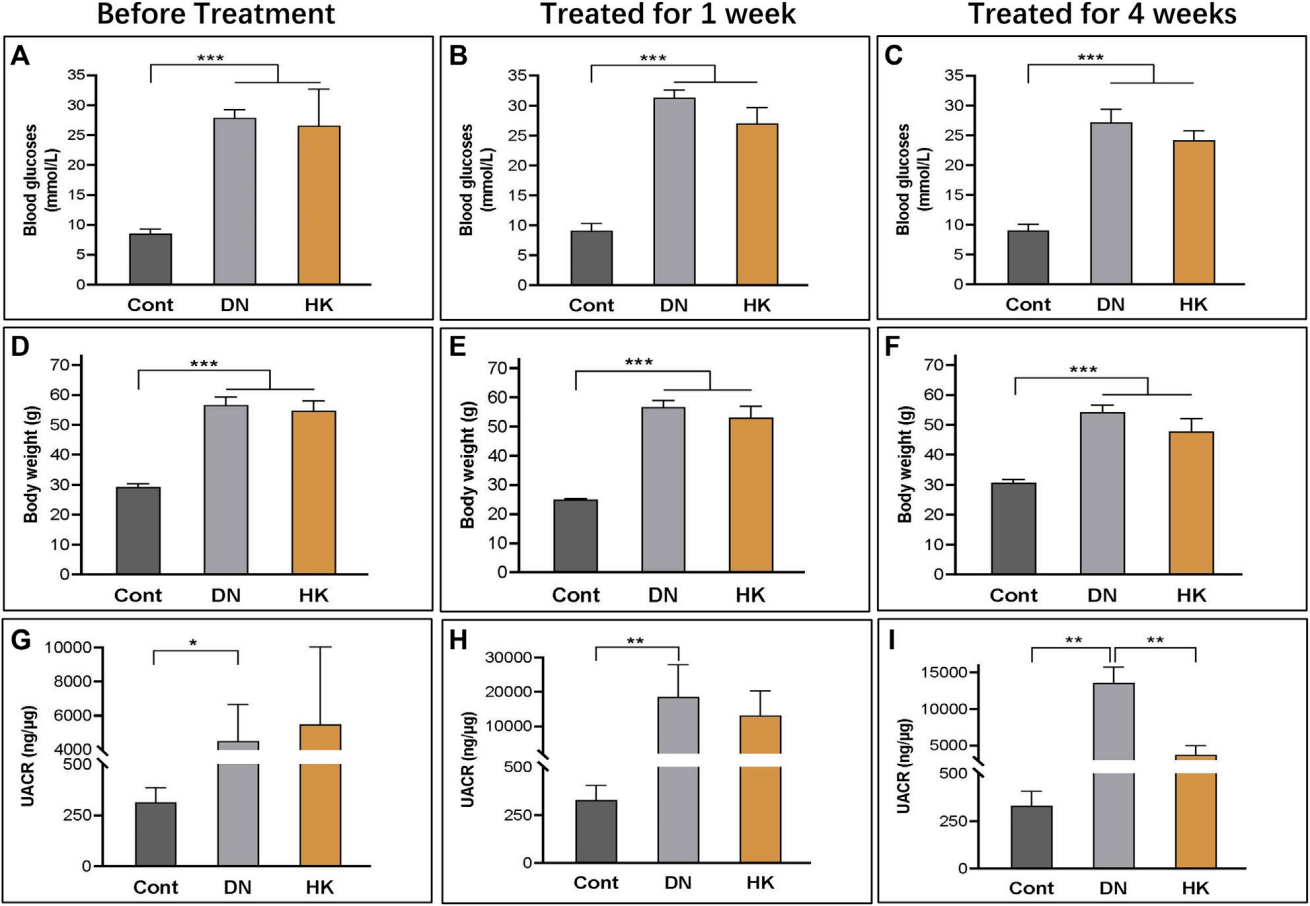
Changes in blood glucose levels, body weight, and the UACR before and after HK capsule treatment. The body weight (**A–C**), blood glucose levels (**D–F**), and UACR (**G–I**) in non-diabetic controls and db/db mice before and after HK capsule treatment for 1 week and 4 weeks. UACR: urinary albumin-to-creatinine ratio; *P<0.05, **<0.01, and ***<0.001.

The efficacy of HK capsule on the reduction of albuminuria and proteinuria in kidney diseases, including DN, has been well documented (Zhang et al., 2014; Li et al., 2017; Li et al., 2020b; Li et al., 2021; Zhao et al., 2022). In the current study, we examined the body weight and blood glucose levels and measured the UACR weekly before and after treatment with HK capsule. As expected, the UACR in db/db mice after HK capsule treatment for 4 weeks was extremely higher than that in non-diabetic controls. After HK capsule treatment for 4 weeks, the UACR was significantly decreased compared to that in db/db mice either without or with HK capsule treatment for 1 week (Figures 1G–I). The body weight (Figures 1A–C) and blood glucose (Figures 1D–F) levels in db/db mice either with or without HK capsule treatment were higher than those in non-diabetic control mice. Although there was a slight decrease in body weight and blood glucose levels in db/db mice after HK capsule treatment compared to the db/db mice without treatment, it was not statistically significant.

### 3.2 Comparison of the renal index and histopathological changes of the kidneys

**FIGURE 2:**
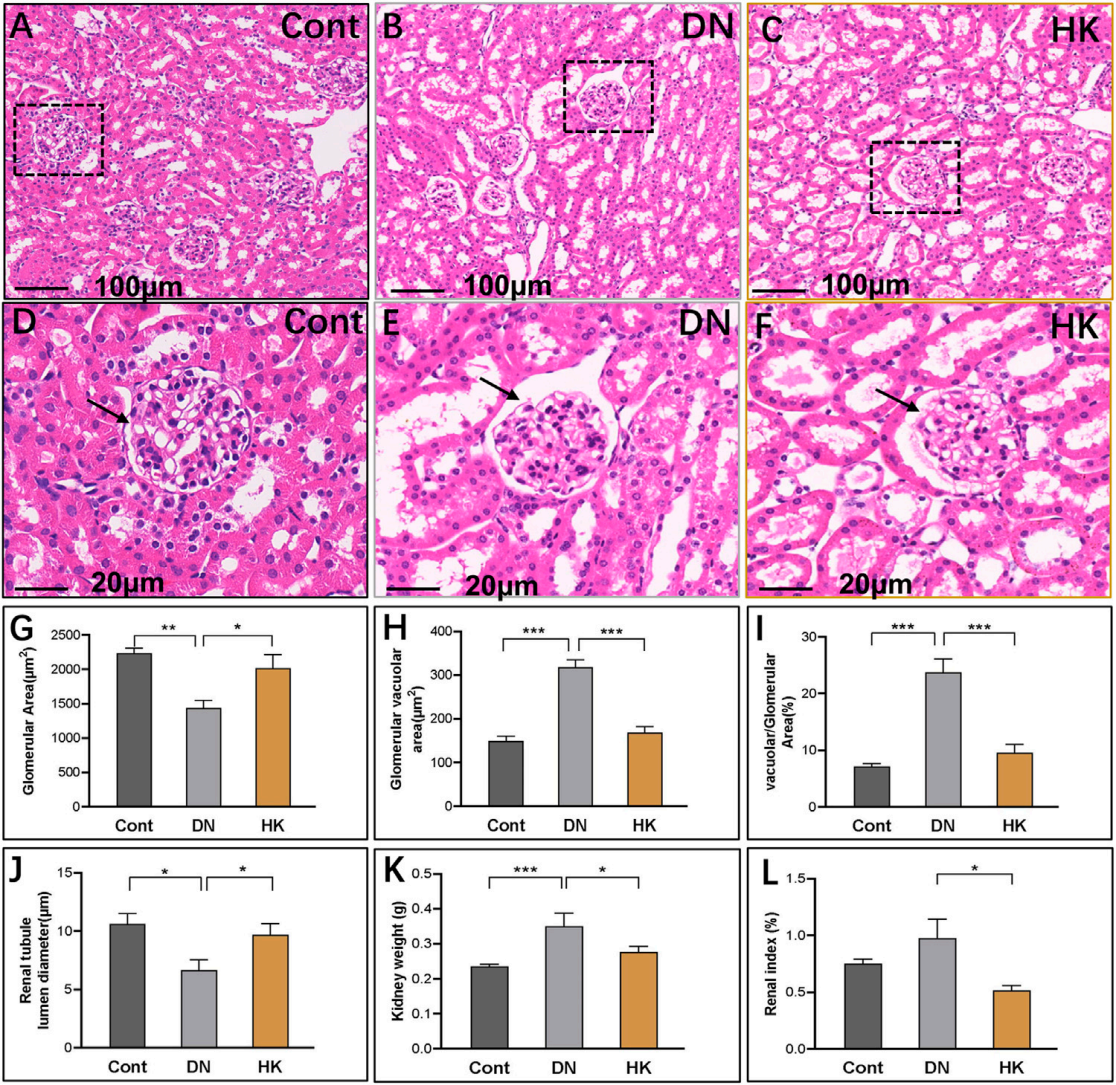
Comparison of the renal index and histopathological changes of the kidneys. H&E staining images of kidney tissue sections in the mice of Cont, DN, and HK groups are represented **(A–F)**. Comparative semi-quantification analyses of the glomerulus, renal tubule lumen diameter, and vacuole area in the mice of Cont, DN, and HK groups are summarized **(G–J)**, and the kidney weight and renal index are shown **(K, L)**, respectively. H&E: hematoxylin and eosin; Cont: non-diabetic control mice; DN: db/db mice with diabetic nephropathy; HK: db/db mice with Huangkui capsule treatment; renal index: the ratio of kidney weight and body weight; *P<0.05, **<0.01, and ***<0.001.

By using a standard staining protocol of hematoxylin and eosin (H&E) staining, we performed the histopathological analyses of kidney sections from the mice in Cont, DN, and HK groups. The images implicated that damage to the glomerular structure, loss of renal cells, podocyte deficiency, and other symptoms were seen in the db/db mice of the DN group in comparison with the mice in the Cont group, while these other symptoms were improved after HK capsule treatment (Figures 2A–F). Furthermore, semi-quantification analysis indicated that the glomerulus and vacuole area were increased in the DN group compared with the Cont group but decreased after HK capsule treatment (Figures 2H, I), while the glomerular area and renal tubule lumen diameter were decreased in the DN group compared with the Cont group but increased after HK capsule treatment (Figures 2G, J). We also analyzed the weight of kidneys and renal index (kidney weight/body weight). Data showed that the kidney weight and renal index in the db/db mice of the DN group were increased compared to those in the mice of the Cont group but decreased after HK capsule treatment (Figures 2K, L).

### 3.3 Col4a3 expression in the glomerular basement membrane

**FIGURE 3:**
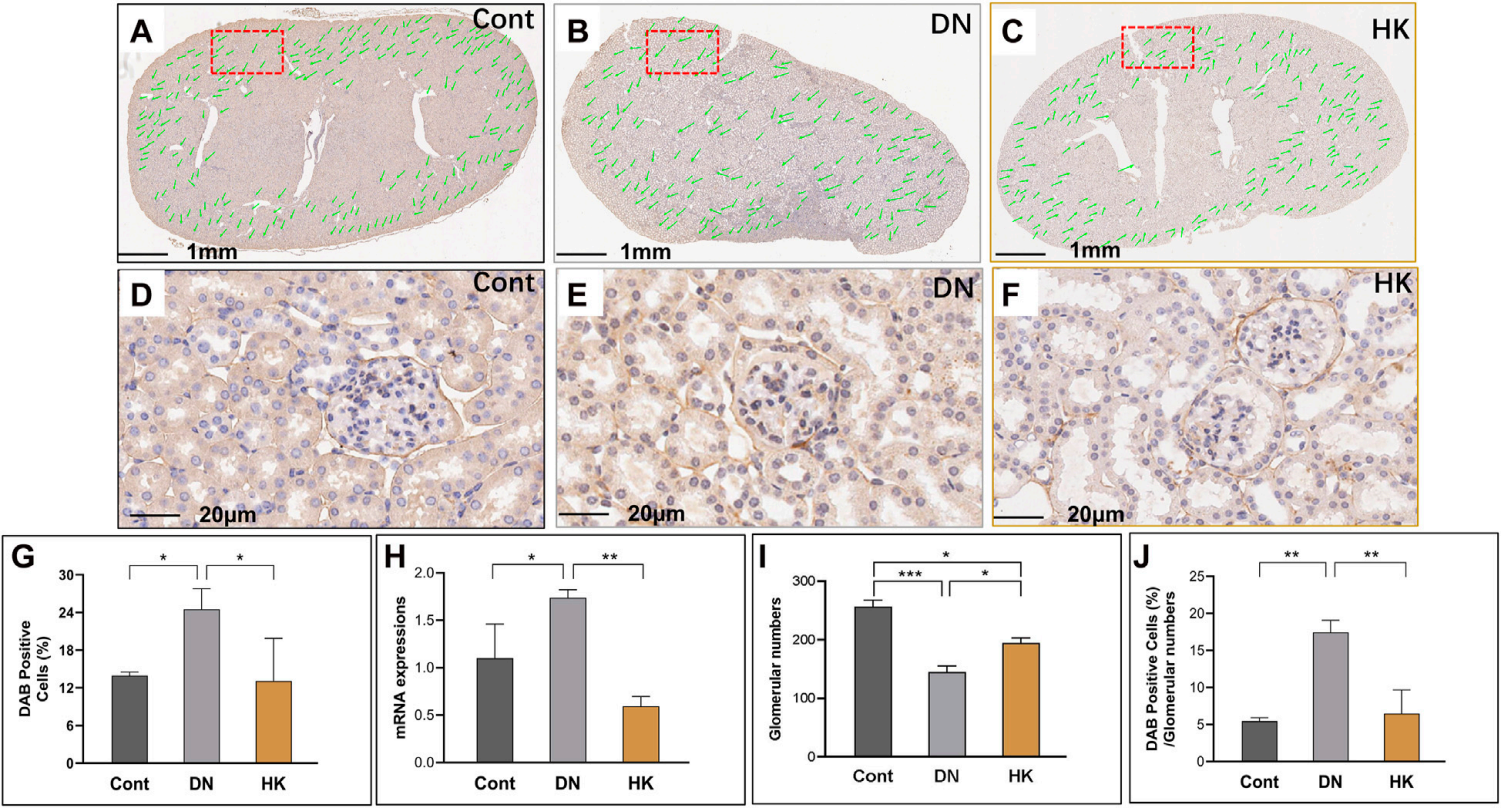
Changes of col4a3 expression in the glomerular basement membrane. Immunochemistry detection of col4a3 in the field of whole kidney sections of the mice in the Cont, DN, and HK groups is represented (**A–C**), and the magnified and randomly selected fields are shown (**D–F**). The col4a3 gene expression at both protein and mRNA levels is summarized (**G, H**). The number of glomeruli in the kidneys (**I**) and DAB-positive cell/glomerular number (**J**) in each group are analyzed comparatively. Cont: non-diabetic control mice; DN: db/db mice with diabetic nephropathy; HK: db/db mice with Huangkui capsule treatment; *P<0.05, **<0.01, and ***<0.001.

Nephron is the basic unit of the kidneys to perform its function. The glomerular filtration barrier is composed of endothelial cells, podocytes, and the GBM, while the GBM is a key component of the glomerular capillary wall and is essential for kidney filtration [8, 9]. Based on its significance in the GBM morphologically and functionally, we selected COL4A3 as a biomarker for the glomeruli in the kidneys. In the current study, we first detected the col4a3 expression in the field of whole kidney sections of the mice in the Cont, DN, and HK groups (Figures 3A–C) and then examined the magnified and randomly selected fields (Figures 3D–F). Data showed that the col4a3 gene expression at both protein and mRNA levels (Figures 3G, H) was decreased after HK capsule treatment. Furthermore, the number of glomeruli in the kidney after HK capsule treatment was higher (Figure 3I), while the DAB-positive cell/glomerular number was lower (Figure 3J) compared to that in DN.

### 3.4 Slc5a2 and slc34a1 expressions in the proximal convoluted tubules

**FIGURE 4:**
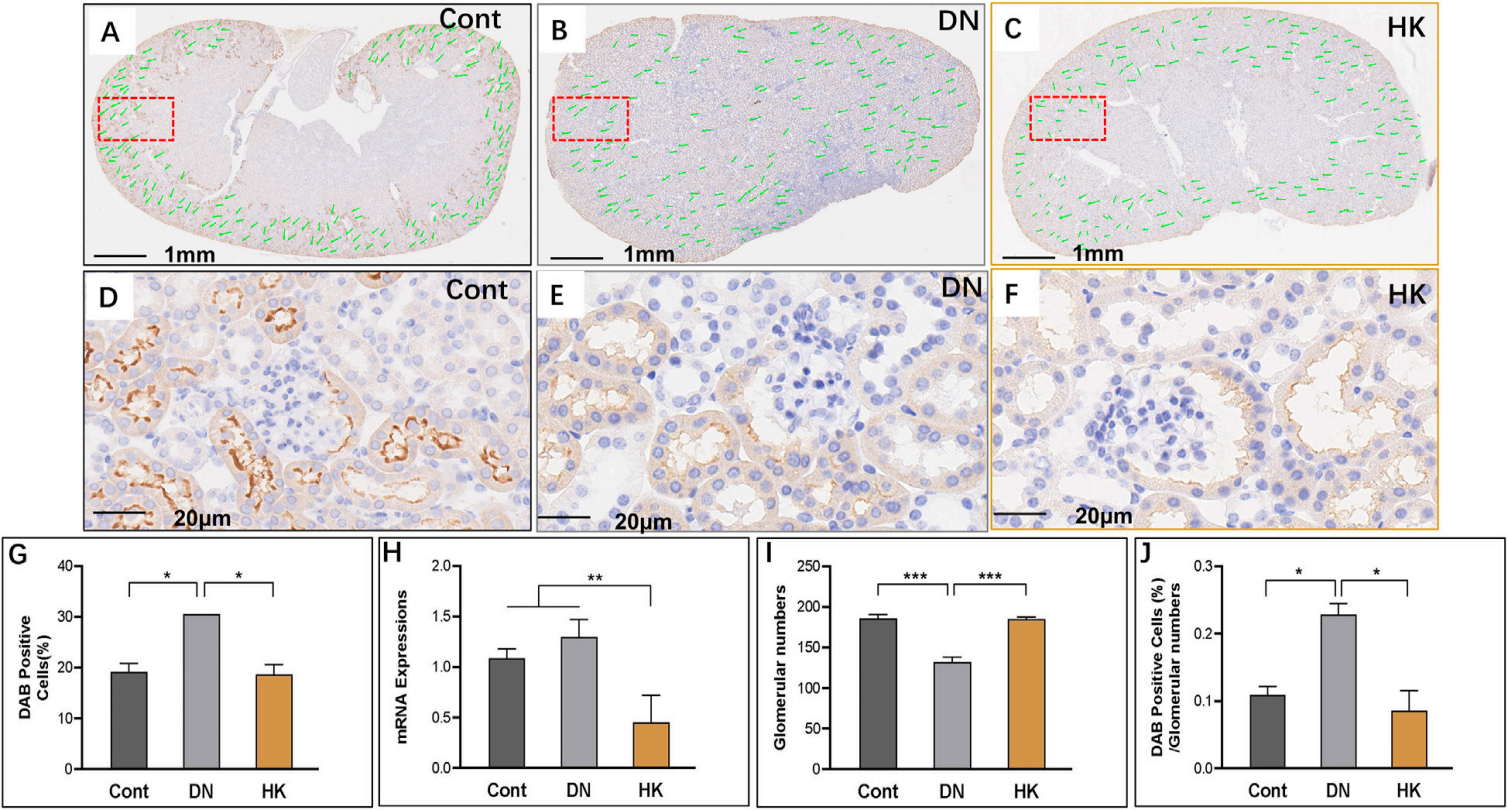
Changes of slc5a2 expression in the proximal convoluted tubules. Immunochemistry detection of slc5a2 in the field of whole kidney sections of the mice in the Cont, DN, and HK groups is represented (**A–C**), and the magnified and randomly selected fields are shown (**D–F**). The slc5a2 gene expression at both protein and mRNA levels is summarized (**G, H**). The number of glomeruli in the kidneys (**I**) and DAB-positive cell/glomerular number (**J**) in each group are analyzed comparatively. Cont: non-diabetic control mice; DN: db/db mice with diabetic nephropathy; HK: db/db mice with Huangkui capsule treatment; *P<0.05, **<0.01, and ***<0.001.

SLC5A2 is a well-established marker for the PCT of the kidneys (Park et al., 2018; Ferrada et al., 2022; Wu et al., 2022). In the current study, we found that slc5a2 gene expression in db/db mice with DN was higher than that in control mice but significantly downregulated after HK capsule treatment for 4 weeks (Figures 4G, H). Interestingly, we analyzed the fields of whole kidney sections and found that the glomerular number in db/db mice with DN was lower than that in control mice, while the number was increased after HK capsule treatment (Figure 4I). We further reanalyzed the data by using the index of DAB-positive cell/glomerular number and confirmed that slc5a2 could be downregulated by HK capsule treatment (Figure 4J).

**FIGURE 5:**
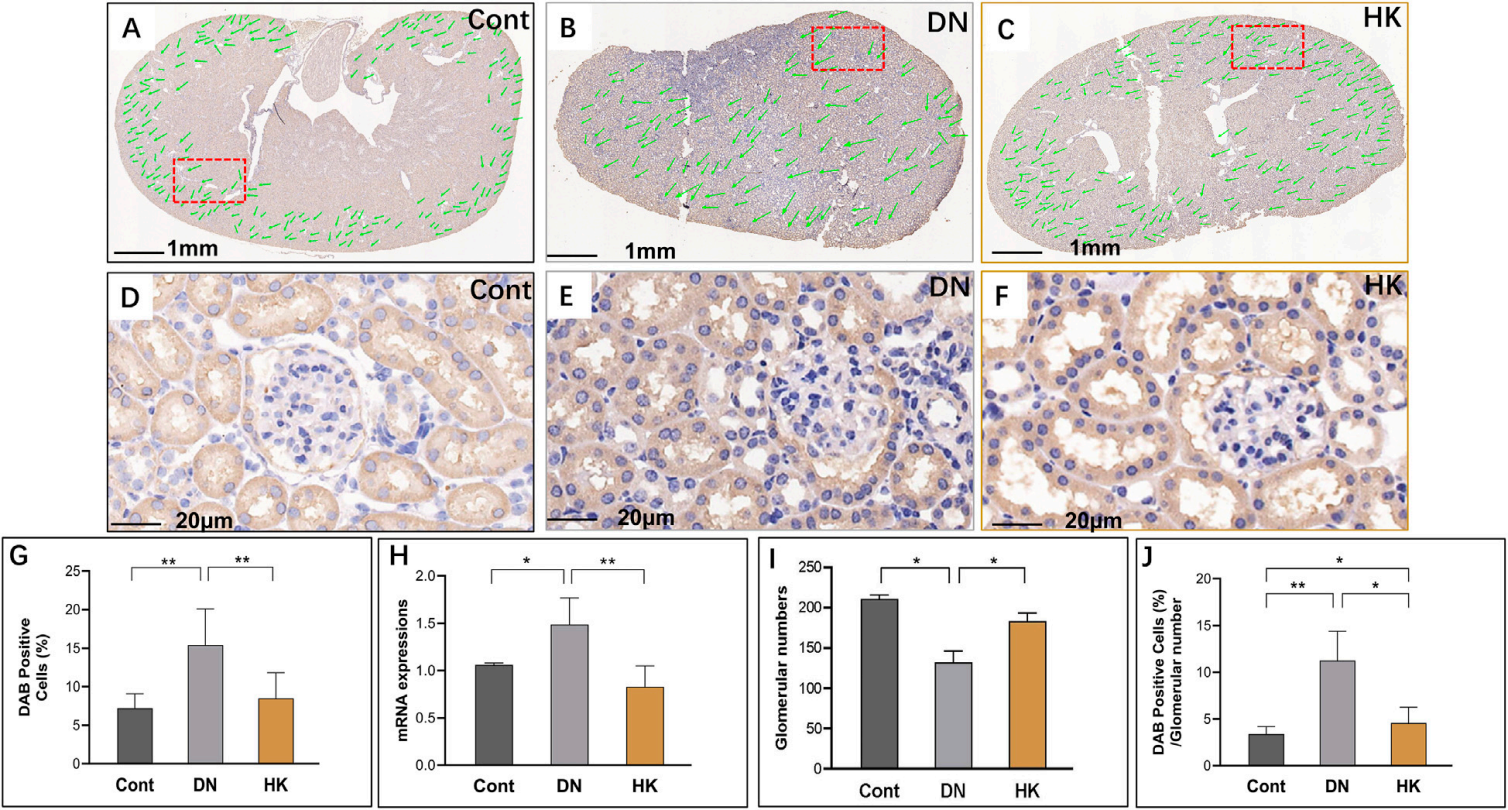
Changes of slc34a1 expression in the proximal convoluted tubules. Immunochemistry detection of slc34a1 in the field of whole kidney sections of the mice in the Cont, DN, and HK groups is represented (**A–C**), and the magnified and randomly selected fields are shown (**D–F**). The slc34a1 gene expression at both protein and mRNA levels is summarized (**G, H**). The number of glomeruli in the kidneys (**I**) and DAB-positive cell/glomerular number (**J**) in each group are analyzed comparatively. Cont: non-diabetic control mice; DN: db/db mice with diabetic nephropathy; HK: db/db mice with Huangkui capsule treatment; *P<0.05, **<0.01, and ***<0.001.

Like SLC5A2, SLC34A1 is also expressed in the PCT of kidneys, and its mRNA expression levels are higher than those in slc5a2 according to the data from Genomic-Tissue Expression (GTEx) in normal human kidney tissues. The *SLC34A1* gene has been considered one of the loci associated with estimated glomerular filtration rate (eGFR) and CKD (Böger et al., 2011). Furthermore, mutations of this gene may cause urolithiasis, which is associated with diabetes (Yasui et al., 2017). We, thus, selected this gene as a biomarker to further evaluate the effects of HK capsule in the proximal convoluted tubules of the kidneys. We found that slc34a1 activities, similar to slc5a2, were increased in db/db mice compared with controls but decreased after HK capsule treatment. In addition, the glomerular number in db/db was reduced in comparison with controls but reversely increased after HK treatment (Figures 5A–J).

### 3.5 Slc12a3 and slc4a1 expressions in distal convoluted tubules

**FIGURE 6:**
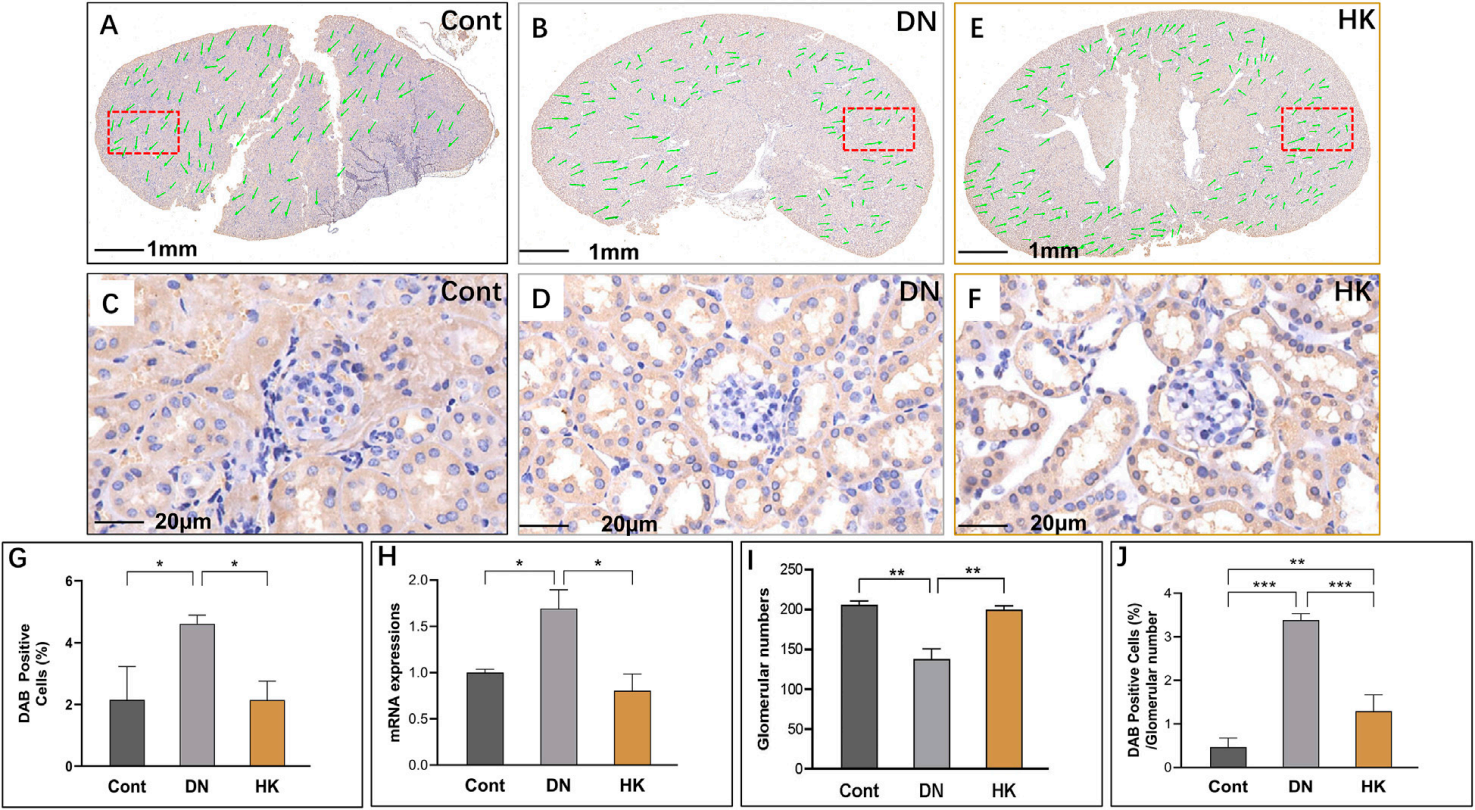
Changes of slc12a3 expression in the distinct convoluted tubules. Immunochemistry detection of slc12a3 in the field of whole kidney sections of the mice in the Cont, DN, and HK groups is represented (**A–C**), and the magnified and randomly selected fields are shown (**D–F**). The slc12a3 gene expression at both protein and mRNA levels is summarized (**G, H**). The number of glomeruli in the kidneys (**I**) and DAB-positive cell/glomerular number (**J**) in each group are analyzed comparatively. Cont: non-diabetic control mice; DN: db/db mice with diabetic nephropathy; HK: db/db mice with Huangkui capsule treatment; *P<0.05, **<0.01, and ***<0.001.

We have previously reported that slc12a3 gene expression is higher in db/db mice at the ages of 6, 12, and 26 weeks in parallel compared to non-diabetic mice at the same ages (Abu Seman et al., 2014). In the current study, we examined slc12a3 as a marker of the DCT in kidneys of db/db mice with and without HK capsule treatment, and images are represented in Figures 6A–G. Similar to what we have previously reported, slc12a3 gene expression at mRNA and protein levels was increased in db/db mice without HK capsule treatment compared to the mice of the Cont group but decreased in db/db mice after HK treatment (Figures 6G, H). Furthermore, the index of DAB-positive cells/glomerular numbers in db/db mice with HK capsule treatment was decreased while the glomerular number was increased compared with the mice without HK treatment (Figures 6I, J).

**FIGURE 7:**
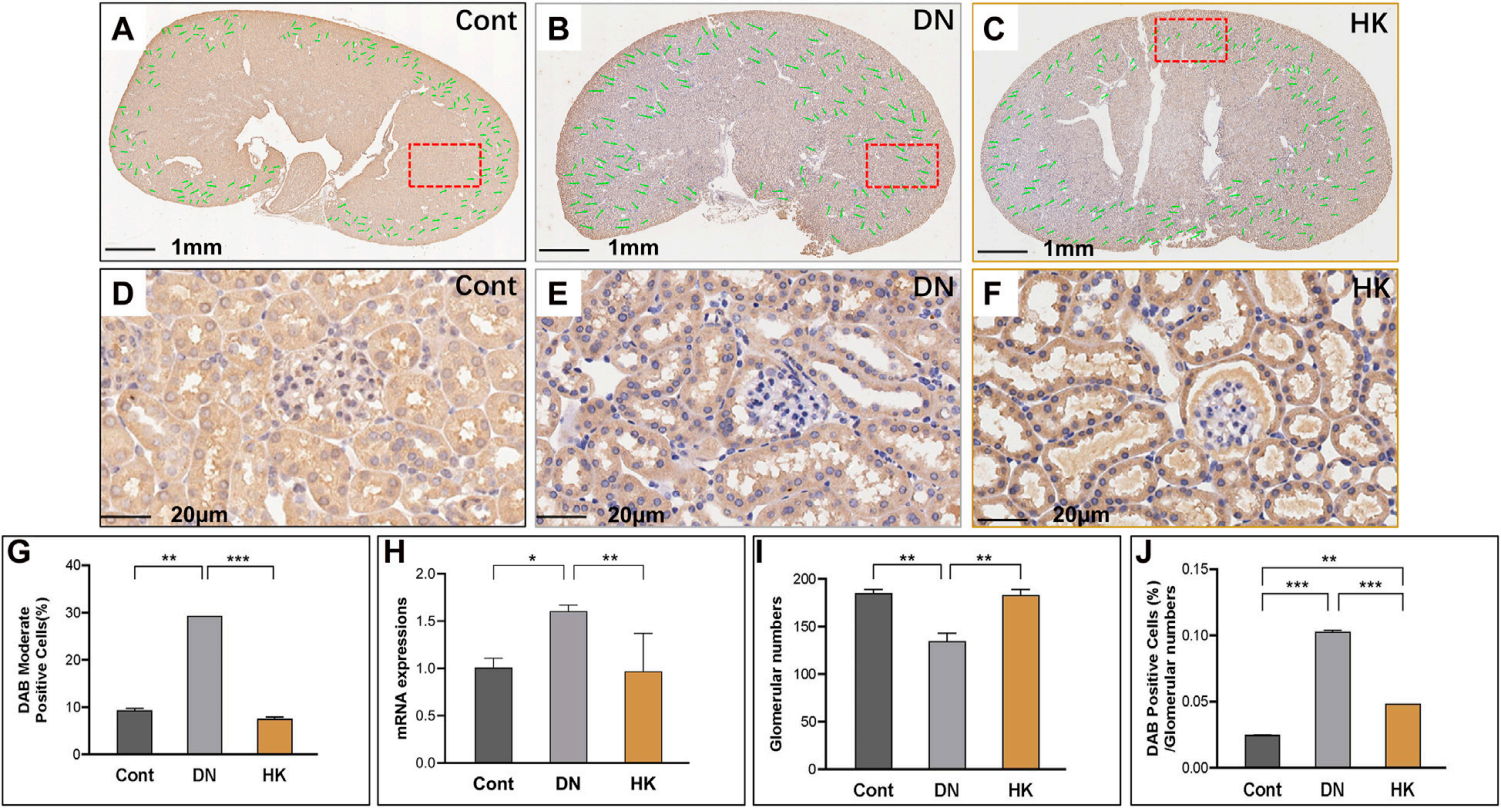
Changes of slc4a1 expression in the distal convoluted tubules. Immunochemistry detection of slc4a1 in the field of whole kidney sections of the mice in the Cont, DN, and HK groups is represented (**A–C**), and the magnified and randomly selected fields are shown (**D–F**). The slc4a1 gene expression at both protein and mRNA levels is summarized (**G, H**). The number of glomeruli in the kidneys (**I**) and DAB-positive cell/glomerular number (**J**) in each group are analyzed comparatively. Cont: non-diabetic control mice; DN: db/db mice with diabetic nephropathy; HK: db/db mice with Huangkui capsule treatment; *P<0.05, **<0.01, and ***<0.001.

*SLC4A1* encodes a membrane protein and is highly expressed in the DCT of kidneys and relatively abundant in the lung and red blood cells. As the product of *SLC4A1*, anion exchanger 1 exchanges extracellular bicarbonate (HCO_3_^−^) for intracellular chloride (Cl^−^) and participates in acid–base homeostasis (Bertocchio et al., 2020). We detected slc4a1 expression at mRNA and protein levels in db/db mice with and without HK capsule treatment and found that slc4a1 expression was downregulated in db/db after HK capsule treatment, although its activity in db/db mice was increased compared with non-diabetic controls (Figures 7A–J).

## 4 Discussion

**FIGURE 8:**
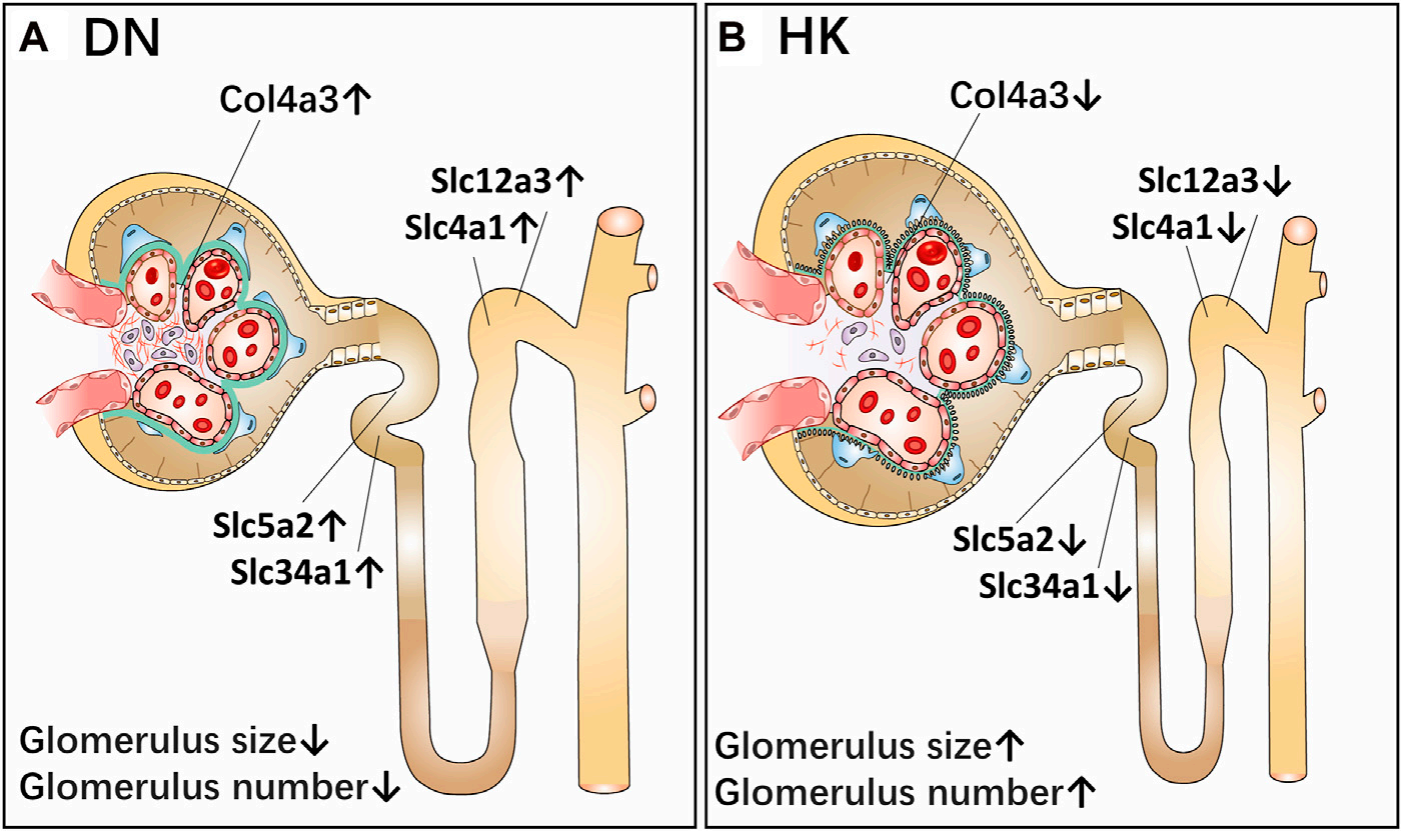
Schematic diagram of the effects of *Abelmoschus manihot* (L.) on diabetic nephropathy via the reregulation of biomarkers in the glomeruli and proximal and distal convoluted tubules of the kidneys. As a single medicament of traditional Chinese medicine*, Abelmoschus manihot* (L.) in the form of HK capsule has been used to treat kidney diseases, including DN. The most significant effect of HK capsule treatment in kidney diseases is the reduction of albuminuria and proteinuria. The current study aims to evaluate the efficacy of HK capsule in the regression of DN by using db/db mice, an animal model for T2D and DN. After HK capsule treatment for 4 weeks, the urine albumin-to-creatinine ratio in db/db mice was found to be significantly decreased. Furthermore, in the kidneys of db/db mice with DN, the structure of the glomeruli of the kidneys in db/db mice was damaged, and the size and number of glomeruli were less than those in non-diabetic mice. Col4a3 in the glomerulus, slc5a2 and slc34a1 in the PCT, and slc12a3 and slc4a1 in the DCT of kidneys are upregulated (**A**). After HK capsule treatment, however, the damage in the kidneys is reduced while the size and number of glomeruli are increased. Col4a3 in the glomerulus, slc5a2 and slc34a1 in the PCT, and slc12a3 and slc4a1 in the DCT of kidneys are downregulated (**B**). DN: diabetic nephropathy; T2D: type 2 diabetes; PCT: proximal convoluted tubule; DCT: distal convoluted tubule; HK: Huangkui capsule.

In the current study, we first confirmed that HK capsule treatment could reduce the UACR and proteinuria in db/db mice and then comparatively analyzed the changes of biological activities of five biomarkers in the nephron of kidneys before and after HK capsule treatment by using db/db mice, an animal model for the study of T2D and DN. These biomarkers are col4a3, slc5a2, slc34a1, slc12a3, and slc4a1, and they are located in the glomerulus and proximal and distal convoluted tubules of kidneys. Data from our study implicate that col4a3, slc5a2, slc34a1, slc12a3, and slc4a1 are upregulated in DN. After HK capsule treatment, however, their activities are downregulated. A schematic diagram of the effects of *A. maniho*t (L.) on DN via the reregulation of these biomarkers in the glomeruli and proximal and distal convoluted tubules of the kidneys is represented in Figure 8.

By using the approach of GWAS, Salem et al. demonstrated that *COL4A3* is a susceptibility gene for DN. The missense mutation rs55703767 (Asp326Tyr) in this gene is significantly associated with DN. Furthermore, the minor allele (326Tyr) in *COL4A3* is found to be protective against several definitions of DN, including albuminuria and ESRD, while the strongest protective effect was observed in patients with glycemia (Salem et al., 2019). This protective effect can be explained by a hypothesis as either the variant confers tensile strength or flexibility to the glomerular basement membrane (GBM). The variant of *COL4A3* may regulate the rates of production and/or turnover of other GBM components, affecting the GBM width changes in diabetes (Pieri et al., 2020). Moreover, genetic mutations in the genes encoded by other chains of type IV collagen are found to be associated with a range of hereditary human kidney diseases, such as Alport syndrome and DN (Jarad et al., 2016). In the current study, we examined col4a3 in the specific location of the GBM and found this gene to be overexpressed in db/db mice. After HK capsule treatment, however, the expressive levels of col4a3 were decreased, which may implicate that the functional defect of col4a3 is involved in the pathogenesis of DN. The downregulation of col4a3 may be one of the mechanisms for *A. Manihot* in DN medication.

Kidney hypertrophy is a common clinical feature in patients with T2D and DN. T2D and arterial hypertension are major cardiovascular risk factors that share metabolic and hemodynamic abnormalities and pathophysiological mechanisms. High sodium intake was found to be associated with increased blood pressure. Slc5a2 is specifically expressed in the PCT of the kidneys and functionally acts as a sodium–glucose cotransporter. The epithelial proliferation accompanied by SLC5A2 upregulation, rather than cellular hypertrophy, has been found to predominantly occur in the hypertrophic kidney in both T1D and T2D (Berra et al., 2020; Lai et al., 2022). In the recent years, SGLT2 inhibitors have been widely used to treat patients with T2D-DN owing to their important benefits in controlling blood glucose levels, reducing cardio-cerebral vascular events and kidney outcomes in patients with diabetes and severe albuminuria (Uehara-Watanabe et al., 2022). In the current study, we observed that body weight and blood glucose levels of db/db mice were slightly, but not significantly, decreased after 4 weeks of HK capsule treatment, as previously reported (Kim et al., 2018), because HK capsule, as a TCM, has multiple pharmacological targets. The effect of HK capsule on the target of Slc5a2 is weaker than that of specialized drugs, i.e., SGLT2 inhibitors.

Slc12a3 is a well-established marker in the DPC of kidneys (Abu Seman et al., 2014; Park et al., 2018; Li et al., 2021; Wu et al., 2022). We have demonstrated that with the knockdown of zebrafish ortholog, slc12a3 led to structural abnormality of the kidney pronephric distal duct at the 1-cell stage. Among db/db mice at the ages of 12 w (T2D) and 26 w (DN), the expression levels of slc12a3 in kidney tissues were found to be gradually increased (Abu Seman et al., 2014). Furthermore, in the same study, a mutation rs11643718 (Arg913Gln) in the *SLC12A3* gene was found to be associated with DN in a Malaysian population (Abu Seman et al., 2014). The association between the *SLC12A3* Arg913Gln variant and T2D-DN was also reported in Japanese and Korean populations (Tanaka et al., 2003; Nishiyama et al., 2005; Kim et al., 2006). Recently, Huang et al., 2022 reported that *SLC12A3* polymorphisms are associated with hypertensive nephropathy in a Chinese cohort. In the current study, we further uncovered that slc12a3 was overexpressed in the DPC of the kidneys in db/db mice with DN while the gene activity could be downregulated after HK capsule treatment. Furthermore, slc34a1 and slc4a1 are two biomarkers in the PCT and DCT of the kidneys, respectively. We found that the activities of these two biomarkers, similar to that in slc5a2 and slc12a3, were upregulated in the kidneys of db/db mice but downregulated after HK capsule treatment.

We examined the activities of five biomarkers in the kidneys of db/db mice before and after HK capsule treatment. To better interpret the results from the current study, we analyzed these biomarkers in whole kidney tissue sections. By examining the whole kidney tissue sections, the structure and number of glomeruli can be identified and then accounted by using computer software and manually as well. Interestingly, we found that the structure of the glomeruli of the kidneys in db/db mice was damaged. The size and number of glomeruli were less than those in non-diabetic mice. After HK capsule treatment, however, the damage in the kidneys of db/db mice was reduced while the size and number of glomeruli were relatively increased. Taken together, the data from the current study not only implicate that the development of DN has been regressed after HK capsule treatment but also suggest that there may be a significance of HK capsule medication in the early stage of DN.

There are a couple of limitations in the current study. First, we carried out the experiments with HK capsule but not with the chemical constituents of *A. maniho*t (L.). Previous studies with chromatographic and spectroscopic analyses demonstrated that the bioactive constituents in the flower of *A. maniho*t (L.) mainly include flavonoids, such as rutin, hyperoside, hibifolin, isoquercetin, myricetin, quercetin, and quercetin-3-O-robinobioside (Lai, 2006; Lai, 2009; Guo et al., 2015). Investigation of the pharmacological efficacy of total flavonoids in *A. manihot* (L.) has been taken into our consideration. Second, we successfully analyzed slc5a2, slc34a1, slc12a3, and slc4a1 protein expression in whole kidney sections with immunohistochemical staining and HALO analyses but not Western blotting. Based on the genome databases and the recent single-cell transcriptomic analyses of the kidneys in mice from our and other groups, there are approximately 400 and 360 members of SLC families in human and mouse genomes, respectively, while most of them are expressed in the kidney (Park et al., 2018; Schaller and Lauschke, 2019; Wang et al., 2020; Ferrada et al., 2022; Wu et al., 2022). Among SLCs, there are high homologies, and no monoclonal antibodies are commercially available. We analyzed slc5a2, slc34a1, slc12a3, and slc4a1 membrane protein structures. The size, molecular mass, and IDs of slc5a2, slc34a1, slc12a3, and slc4a1 are summarized in Supplementary Figure S1. Clearly, these molecules have a large molecular weight (68937-113139 Da) and shuttle back and forth 11–14 times in the cell membrane. Thereby, the issues of homogenizing kidney tissues and high homologies of these molecules could be the explanation for the difficulty in Western blotting analysis. In recent years, SLCs have been increasingly acknowledged as key biopharmaceutical targets (Lin et al., 2015; Colas et al., 2016). To our knowledge, the current study is the first report to evaluate the effects of *A. maniho*t (L.) in the regulation of slc5a2, slc34a1, slc12a3, and slc4a1. However, our knowledge concerning the changes of SLCs in the pathogenesis of DN and the regulatory roles of *A. maniho*t (L.) in SLCs is still limited. Therefore, in future research on molecular mechanisms of *A. maniho*t (L.) in the treatment of DN, we may need to pay more attention to the study of the functional regulation of the bioactive constituents in *A. maniho*t (L.) on renal tubular epithelial cells, especially for targets such as SLCs.

## 5 Conclusion

The current study provides experimental evidence that *A. manihot* (L.) may have pharmacological efficacy in the regression of DN development and suggests that the application of HK capsule in the early stage of DN may have a better rehabilitation effect for DN patients.

## Data Availability

The original contributions presented in the study are included in the article/Supplementary Material; further inquiries can be directed to the corresponding authors.

## Abbreviations

*A. manihot* (L.), *Abelmoschus manihot* (L.); Col4a3, collagen type IV alpha 3 chain; DN, diabetic nephropathy; DCT, distinct convoluted tubules; ESRD, end-stage renal disease; GWAS, genome-wide association study; HK capsule, Huangkui capsule; PCT, proximal convoluted tubule; Slc12a3, solute carrier family 12 member 3; Slc4a1, solute carrier family 4 member 1; Slc5a2, solute carrier family 5 member 2; Slc34A1, solute carrier family 34 member 1; TCM, traditional Chinese medicine; T1D, type 1 diabetes; T2D, type 2 diabetes; and UACR, urinary albumin-to-creatinine ratio.
