# Supplementary material for: Evaluation of the efficacy of Abelmoschus manihot (L.) on diabetic nephropathy by analyzing biomarkers in the glomeruli and proximal and distal convoluted tubules of the kidneys
Source: Front Pharmacol. 2023 Aug 1;14:1215996. doi: 10.3389/fphar.2023.1215996 (PMC10427220; doi:10.3389/fphar.2023.1215996)
Supplement: Supplementary file 1 [file DataSheet1.PDF]

Supplemental Table 1 Selection of biomarkers in glomerulus, proximal and distal convoluted tubules of kidneys

Gene symbol	Full name/Aliases	Genomic location*/Cytogenetic band	Protein accession, size & molecular mass	REFSEQ mRNA (GTEx)	Expressed in kidneys	Biological function	Orthologs#	Reference
COL4A3	Collagen Type IV Alpha 3 Chain	chr2:227,164,624-227,314,792<br>2q36.3	Q01955<br>1670AA<br>161813 Da	NM_000091.5<br>Highly expressed in kidneys (47)	Glomerular basement	A major structural component of basement membranes	81.49 (n)	Salem RM et al. 2019
SLC5A2	Solute Carrier Family 5 (Sodium/Glucose Cotransporter), Member 2/SGLT2	chr16:31,482,535-31,490,860<br>16p11.2	P31639<br>672 AA<br>72897 Da	NM_003041.4<br>Highly expressed in kidneys (57)	Proximal tubules	A major cotransporter in glucose and sodium reabsorption	84.75 (n)	Alicic R et al. 2018 & 2019
SLC34A1	Solute Carrier Family 34 (Type II Sodium/Phosphate Cotransporter), Member 1	chr5:177,379,235-177,412,021<br>5q35.3	Q06495<br>639 AA<br>68937 Da	NM_001167579.2<br>NM_003052.5<br>Highly expressed in kidneys (70)	Proximal tubules	A transporter of phosphate into cells via sodium cotransport in the renal brush border membrane	86.24 (n)	Köttgen A et al. 2010
SLC12A3	Solute Carrier Family 12 (Sodium/Chloride Transporter), Member 3	chr16:56,865,207-56,915,850<br>16q13	P55017<br>1021 AA<br>113139 Da	NM_000339<br>Highly expressed in kidneys (71)	Distal convoluted tubules	A renal thiazide-sensitive sodium-chloride cotransporter and is important for electrolyte homeostasis	87.08 (n)	Abu Seman N et al. 2014
SLC4A1	Solute Carrier Family 4 Member 1 (Diego Blood Group)	chr17:44,248,390-44,268,141<br>17q21.31	P02730<br>911AA<br>101792 Da	NM_000342.4<br>Highly expressed in kidneys (45)	Distal convoluted tubules	A chloride/bicarbonate exchanger involved in carbon dioxide transport from tissues to lungs.	82.65 (n)	Trepiccione F et al. 2017; Bertocchio JP et al. 2020

Data are collected from GeneCards and PubMed; AA: amino acid; GTEx: mRNA Expression levels in normal human kidney tissues; *Latest assembly from GRCh38/hg38; #Similarity between human and mouse.

Supplemental Table 2 The primers used in real time RT-PCR

**Gene**	**Forward primer**	**Reverse primer**
col4a3	5’- CGGTGTCAGAGGTGATCCAG-3’	5’-CCACGTGGACCTGGTGGT-3’
slc5a2	5’-TGGTGTTGGCTTGTGGTCTA-3’	5’-ATGTTGCTGGCGAACAGAGA-3’
slc34a1	5’- GGGCTCCAACATTGGCACTA-3’	5’-GGGCTCCAACATTGGCACTA-3’
slc12a3	5’-GACAGGCACCAACAGTGAGA-3’	5’-GCATGCAACGGATCATCACC-3’
slc4a1	5’-GGGCTCCAACATTGGCACTA-3’	5’-TGGCAGAGGGCAATCTGAAAT-3’
gapdh	5’- GGGTCCCAGCTTAGGTTCAT-3’	5’-CCCAATACGGCCAAATCCGT-3’

Supplemental Figure 1
Prediction of transmembrane structures of SLCs in human (A) and mouse (B)

A. Human

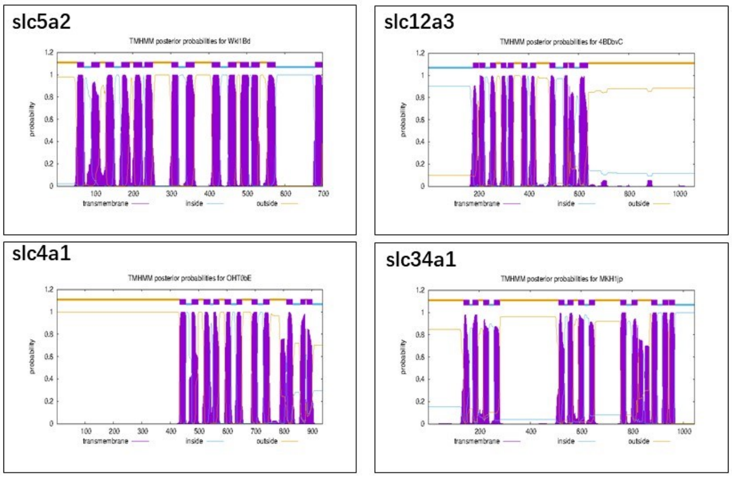

B. Mouse

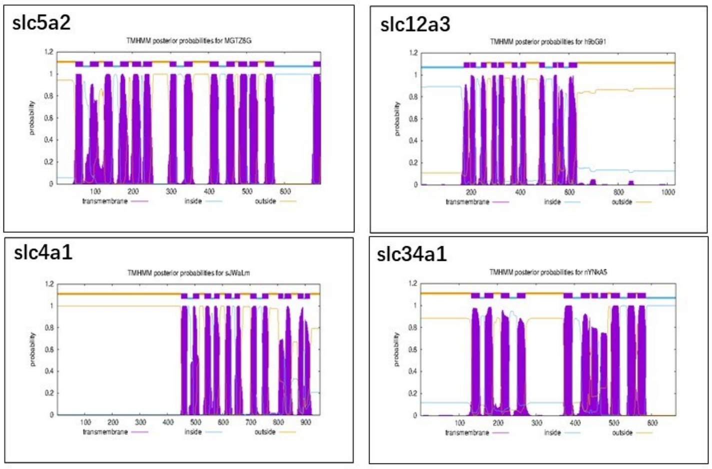

Data are generated by using TMHMM-2.0. Protein size, molecular mass, and IDs of each SLC in human and mouse are summarized as below.
Size:672 amino acids
Molecular mass:72897 Da
>NP_573517.1 Slc5a2 [organism=Mus musculus] [GeneID=246787]
>NP_003032.1 SLC5A2 [organism=Homo sapiens] [GeneID=6524]

Size:639 amino acids
Molecular mass:68937 Da
>NP_035522.2 Slc34a1 [organism=Mus musculus] [GeneID=20505]
>NP_001161051.1 SLC34A1 [organism=Homo sapiens] [GeneID=6569] [isoform=2]

Size:1021 amino acids
Molecular mass:113139 Da
>NP_001192240.1 Slc12a3 [organism=Mus musculus] [GeneID=20497] [isoform=1]
>NP_000330.3 SLC12A3 [organism=Homo sapiens] [GeneID=6559] [isoform=1]

Size:911 amino acids
Molecular mass:101792 Da
>NP_035533.1 Slc4a1 [organism=Mus musculus] [GeneID=20533]
>NP_000333.1 SLC4A1 [organism=Homo sapiens] [GeneID=6521]
